# Bnip3 expression is strongly associated with reelin-positive entorhinal cortex layer II neurons

**DOI:** 10.1007/s00429-024-02816-1

**Published:** 2024-06-25

**Authors:** Stig W. Omholt, Raissa Lejneva, Maria Jose Lagartos Donate, Domenica Caponio, Evandro Fei Fang, Asgeir Kobro-Flatmoen

**Affiliations:** 1 Department of Circulation and Medical Imaging, Norwegian University of Science and Technology (NTNU), 7491 Trondheim, Norway; 2 Kavli Institute for Systems Neuroscience, Norwegian University of Science and Technology (NTNU), 7491 Trondheim, Norway; 3 K. G. Jebsen Centre for Alzheimer’s Disease, Norwegian University of Science and Technology (NTNU), 7491 Trondheim, Norway; 4 Department of Clinical Molecular Biology, University of Oslo and Akershus University Hospital, 1478 Lørenskog, Norway

**Keywords:** Neurometabolism, Synaptic modulation, Neuroanatomical markers, Mitophagy

## Abstract

In layer II of the entorhinal cortex, the principal neurons that project to the dentate gyrus and the CA3/2 hippocampal fields markedly express the large glycoprotein reelin (Re + ECLII neurons). In rodents, neurons located at the dorsal extreme of the EC, which border the rhinal fissure, express the highest levels, and the expression gradually decreases at levels successively further away from the rhinal fissure. Here, we test two predictions deducible from the hypothesis that reelin expression is strongly correlated with neuronal metabolic rate. Since the mitochondrial turnover rate serves as a proxy for energy expenditure, the mitophagy rate arguably also qualifies as such. Because messenger RNA of the canonical promitophagic BCL2 and adenovirus E1B 19-kDa-interacting protein 3 (Bnip3) is known to be highly expressed in the EC, we predicted that Bnip3 would be upregulated in Re + ECLII neurons, and that the degree of upregulation would strongly correlate with the expression level of reelin in these neurons. We confirm both predictions, supporting that the energy requirement of Re + ECLII neurons is generally high and that there is a systematic increase in metabolic rate as one moves successively closer to the rhinal fissure. Intriguingly, the systematic variation in energy requirement of the neurons that manifest the observed reelin gradient appears to be consonant with the level of spatial and temporal detail by which they encode information about the external environment.

**Supplementary Information:**

The online version contains supplementary material available at 10.1007/s00429-024-02816-1.

## Introduction

The entorhinal cortex (EC), which in humans is located deep in the medial temporal lobe, processes an enormous amount of information, mediating a large majority of the information flow between the neocortex and the hippocampus (Cappaert et al. 2015). Functionally, EC plays a crucial role in the formation, consolidation, and retrieval of memories and spatiotemporal representations (Sugar and Moser 2019). The definition of EC is anchored to the fact that it projects to the hippocampal dentate gyrus, aligned with specific cytoarchitectonic features. In rodents, two main subdivisions are easily recognized, namely the medial EC (MEC) and the lateral EC (LEC) (Kobro-Flatmoen and Witter 2019). These subdivisions have their counterparts in primates, including humans, in which the anteriolateral EC approximately corresponds to LEC, and the posteromedial EC approximately corresponds to MEC (Kobro-Flatmoen and Witter 2019; Syversen, et al. 2022; Navarro Schroder et al. 2015; Maass et al. 2015). Since, in particular, the population of large clustered projection neurons in the lateral or anterolateral EC layer II (LII) shows clear signs of degeneration from the earliest stages of Alzheimer’s disease (AD) (Kordower et al. 2001; Gomez-Isla et al. 1996; Braak and Braak 1991; Kulason et al. 2019), this domain of ECLII is clearly involved in the very early etiology of the disease (Kobro-Flatmoen et al. 2021; Ramsden et al. 2023). Therefore, a better understanding of native ECLII physiology is of biomedical interest.

In the adult brain, the large glycoprotein reelin functions as a potent inducer of synaptic spine-growth (Qiu et al. 2006) and plasticity (Rogers et al. 2011), and promotes long-term potentiation (Weeber et al. 2002). Reelin is mainly expressed in a subset of interneurons found throughout the cortex. However, in ECLII, one of the two main populations of principal neurons expresses a high level of reelin (Re + ECLII neurons), and these neurons are situated in the superficial half of the layer. The other main population of ECLII principal neurons expresses Calbindin (Kitamura et al. 2014) (Cb + ECLII neurons), and these are situated in the deep half of the layer. It is important to note that a substantial population of EC layer III neurons are also Cb + , and that parallel use of the historical vs the more modern delineation of superficial EC layers can potentially generate some confusion. Specifically, of the Cb + neurons that by the more modern delineation scheme are designated to belong to layer II in rats, a few are intermingled with the Re + population while most are situated in the deep part of the layer (sometimes referred to as EC LIIb). These Cb + ECLIIb neurons were originally designated to belong to superficial parts of layer III, such that layer III consisted of a Cb + population in its superficial half and a Cb + population in its deep half, and this scheme of delineation is still in use (Kobro-Flatmoen and Witter 2019). Here we use the more modern separation of the superficial EC layers, meaning that layer II includes superficially located Re + neurons and deeply located Cb + neurons.

The Re + and the Cb + populations also differ with respect to their downstream targets. Re + ECLII neurons from both MEC and LEC project to the dentate gyrus and the CA3/2 hippocampal fields. On the contrary, the downstream targets of Cb + ECLII neurons differ greatly between MEC and LEC, as those of MEC primarily target field CA1, while those of LEC target multiple extra-hippocampal forebrain structures (Ohara et al. 2019).

The Re + ECLII neurons express reelin in a strikingly graded pattern. In rodents, neurons located at the dorsal extreme of EC, that is, at the border of the rhinal fissure, express the highest levels, and the expression gradually decreases toward the ventral extreme (Kobro-Flatmoen et al. 2016). Correcting for the different gross orientation of EC between different orders of mammals, the same pattern holds true for all mammals examined, including humans (Kobro-Flatmoen and Witter 2019).

The morphology of Re + ECLII neurons strongly suggests that they are heavily involved in synaptic transmission. Most Re + ECLII neurons have a stellate (MEC) or fan (LEC) morphology with imposing dendritic trees. A single Re + ECLII-neuron in a rat has a densely spinous dendritic tree whose distal 360 degree diametric perimeter can span upwards of 900 μm (Canto and Witter 2012a, b). To appreciate the size of this span, one may consider another cell type with very large dendritic trees, namely hippocampal pyramidal neurons. These neurons have apical dendritic arbors estimated to be 4–9 times larger and basal dendritic arbors approximately 3 times larger than that of a pyramidal neocortical neuron (Brown et al. 2008). However, the apical (and largest) dendritic arbor of a large pyramidal hippocampal neuron, measured at its distal perimeter (Brown et al. 2008; Andersen et al. 2006), is only about half that of a Re + ECLII-neuron. Likely facilitated by their formidable dendritic trees*, a single* Re + LECLII-neuron is capable of collecting direct input from at least five separate cortical areas, in addition to multiple sources of subcortical modulatory input (Kobro-Flatmoen et al. 2021). Furthermore, these neurons give rise to particularly extensive axonal arborizations both in their downstream hippocampal target zones and locally in EC (Tamamaki and Nojyo 1993). Since most of the brain energy is spent on synaptic transmission (Harris et al. 2012), the energy requirement of these neurons is likely very high. In addition, the presence of high levels of reelin implicates a particularly high capacity for remolding synaptic contacts, adding to their energy requirement. Work on small mammals indicates that reelin is transported along EC axons and released on downstream hippocampal targets (Martinez-Cerdeno et al. 2003). Given the substantial local collateralization of ECLII-axons (Tamamaki and Nojyo 1993) and the fact that a large proportion of ECLII neurons express ApoER2 (Ramsden et al. 2023), reelin is also likely released on local EC targets. Such axonal transport- and release is in line with ultrastructural studies on humans (Roberts et al. 2005), while experiments using primary neurons indicate that reelin might be released via the secretory pathway in a constitutive manner that furthermore involves dendrites (Nakao et al. 2022). In any case, these release processes are likely to be associated with synaptic contact remolding.

The above considerations led us to hypothesize that Re + ECLII neurons in general have a particularly high metabolic rate and that the rate is highest in those located closest to the rhinal fissure. Since mitochondria provide the lion’s share of brain energy (Harris et al. 2012), mitochondrial turnover rate appears to be a superb proxy of metabolic rate under normal physiological conditions as it probably strongly correlates with both the number of mitochondria and the metabolic demand on each mitochondrion, i.e., it correlates with the frequency with which impaired mitochondria arise. Thus, our hypothesis would be supported by establishing that the mitochondrial turnover rate in Re + ECLII neurons is strongly associated with their reelin expression.

The main function of BCL2 and adenovirus E1B 19-kDa-interacting protein 3 (Bnip3) is to induce mitophagy (Bellot et al. 2009; Rikka et al. 2011; Zhang et al. 2016) and the mRNA precursor of Bnip3 is especially enriched in the EC of adult rats (Cho et al. 2012). Bnip3 expression level is arguably positively correlated with the rate of mitophagy, and assuming homeostatic regulation, the rate of mitophagy must in turn be highly correlated with mitochondrial turnover rate. Thus, based on the assumption that Bnip3 expression level can serve as a proxy for cellular energy expenditure, we assayed the relative amount of Bnip3 in the brain of rats. We found that although neurons in nearly all areas contained none or very low levels of Bnip3, which is in line with previous findings (He et al. 2019; Lu et al. 2014; Zhang et al. 2011; Althaus et al. 2006), ECLII constitutes a striking exception. Double-immunofluorescence labeling revealed that nearly every Re + ECLII neuron expresses Bnip3 and that the expression level of Bnip3 aligns with the reelin gradient. Since there appears to be a consonance between the reelin and Bnip3 gradients, how Re + ECLII neurons connect to the hippocampal formation (Kobro-Flatmoen and Witter 2019), and the resolution of encoded environmental representations, we suggest that the systematic variation in energy requirements and reelin expression in Re + ECLII neurons may possibly reflect the frequency by which they need to update information about the external environment.

## Methods

For this study we used eight outbred Wistar Han rats purchased from Charles River (France). We divided the animals into four age-groups, 3-months, 6-months, 12-months, and 18-months, each containing two rats. All procedures were approved by the Norwegian Animal Research Authority, and follow the European Convention for the Protection of Vertebrate Animals used for Experimental and Other Scientific Purposes. We kept the animals on a 12-h light/dark cycle under standard laboratory conditions (19–22 °C, 50–60% humidity), with access to food and water ad libitum. The human case was from archival material obtained from King’s College London Biobank.

*Processing and immunohistochemistry.* We fully anaesthetised the animals by placing them in a chamber containing 5% isoflurane gas (Baxter AS, Oslo, Norway), followed by an intraperitoneal injection of pentobarbital (0.1 ml per 100 g; SANIVO PHARMA AS, Oslo, Norway). Subsequently we carried out transcardial perfusions with Ringer’s solution at room temperature (in mM: NaCl, 145; KCl, 3.35; NaHCO3, 2.38; pH ~ 6.9) immediately followed by circulation of 4% freshly depolymerised paraformaldehyde (PFA; Merck Life Science AS/Sigma Aldrich Norway AS, Oslo, Norway) in a 125 mM phosphate buffer (PB) solution (VWR International, Pennsylvania, USA), pH 7.4. The brains were extracted and post-fixed overnight in PFA at 4 °C, and then placed in a freeze protective solution (dimethyl sulfoxide in 125 mM PB with 20% glycerol, Merck Life Science AS/Sigma Aldrich Norway AS, Oslo, Norway) until sectioning. We sectioned the brains coronally at 40 μm into six series, using a freezing sledge microtome.

Immunohistochemistry was done on free-floating sections. To improve access to the antigen, the sections were subjected to gentle heat induced antigen retrieval by placement in PB at 60 °C for two hours. For chromogenic immunoenzyme-labelling: To avoid unspecific labelling the sections were first incubated for 2 × 10 min in a solution of PB with 3% hydrogen peroxide (Merck Life Science AS/Sigma Aldrich Norway AS, Oslo, Norway), and next incubated for 1 h in a solution of PB with 5% normal goat serum (Abcam Cat# ab7481, RRID: AB_2716553). Subsequently, free-floating sections were incubated with the primary antibody, rabbit anti-BNIP3 (BioNordika, Oslo, Norway, Cat# CST-3769S, RRID:AB_2259284) in a 1:500 dilution in PB containing 0.4% Saponin (VWR International AS, Bergen, Norway) and 5% normal goat serum, for 48 h. We also tested a primary incubation time of 20 h (i.e. overnight), which gave slightly less overall signal intensity. We opted for 48 h to make sure we did not miss any potentially weakly positive neurons or neuropil. Sections were then rinsed for 3 × 5 min in Tris-buffered saline (50 mM Tris, 150 mM NaCl, pH 8.0) containing 0.4% Saponin (TBS-Sap), and then incubated with biotinylated secondary antibody goat anti-rabbit (1:400; Sigma-Aldrich Cat#B8895, RRID: AB_258649) in TBS-Sap, for 90 min at room temperature. Subsequently, the sections were rinsed in TBS-Sap, and then incubated in Avidin–Biotin complex (ABC, Vector Laboratories Cat# PK-4000, RRID: AB_2336818) for 90 min at room temperature. The sections were then rinsed again, and then incubated in a solution containing 0.67% 3,3ʹ-Diaminobenzidine (Sigma/Merck, Cat# D5905) and 0.024% hydrogen peroxide for 4 min. We safeguarded against potentially subtle differences that can arise from small variations in the time of incubation or the solution with the chromogen by incubating sections from different age groups in parallel, using the same solution of Diaminobenzidine. For immunofluorescence labelling: All tissue was first subjected to Heat Induced Antigen Retrieval, by immersion in PB at 60 °C for 2 h, and then blocking against unspecific labelling was done by placing the tissue in 5% goat serum in PB for 1 h. For reelin and Bnip3, we incubated the sections overnight with primary antibodies (mouse anti-reelin G10 clone (1:1000), Millipore, Cat# MAB5364, RRID:AB_2179313 and rabbit anti-BNIP3 (1:500), BioNordika, Oslo, Norway, Cat# CST-3769S, RRID:AB_2259284) in PB solution containing 0.4% Saponin (VWR, Cat# 27,534.187) and with 5% goat serum at 4 degrees Celsius. The sections were subsequently incubated for two hours with secondary antibodies, including Alexa 546 Goat anti-Mouse (Thermo Fisher, Cat#A11003, RRID: AB_10562732) and Alexa 488 Goat anti-Rabbit (Thermo Fisher Scientific Cat# A-11008, RRID:AB_143165), both at 1:500 in a solution containing 0.4% Saponin and 5% goat serum at room temperature. For Bnip3 and tyrosine hydroxylase (TH) double-immunolabeling, we used the same approach as above with rabbit anti-BNIP3 (1:500) and mouse anti-tyrosine hydroxylase (1:1000, Millipore Cat# MAB318, RRID:AB_2201528). For the triple-labelling experiments (Supplementary Fig. 2) we first incubated sections with the primary antibodies against reelin and Bnip3 overnight, before incubating them for two hours with secondary antibodies as detailed above. The sections were subsequently incubated overnight with primary antibody rabbit anti-calbindin (1:5000, Swant Cat# CB38, RRID:AB_10000340), followed by secondary antibody Alexa 647 Donkey anti-Rabbit (1:500, Thermo Fisher, Cat#A-31573, RRID: AB_2536183) for two hours. The clear segregation of the Bnip3 labelling vs the calbindin labelling means that we can safely assume that the Alexa 488 Goat anti-Rabbit secondary antibody had fully saturated the epitopes of the rabbit anti-Bnip3 primary, such that this primary was not bound by the subsequent application of the Alexa 647 Donkey anti-Rabbit secondary antibody.

Processed tissue was mounted on Superfrost^™^ glass slides (Thermo Fisher Scientific) from a solution of 50 mM tris(hydroxymethyl)aminomethane (Millipore, Cat# 1.08382.1000) with hydrochloric acid, at pH 7.6, and then left to dry overnight before being coverslipped using a mixture of Xylene (VWR International AS, Bergen, Norway) and entellan (Merck KGaA, Cat# 1.07960.0500).

*Imaging and quantification.* All tissue sections were scanned on an automated Zeiss Axio Scan.Z1 system with a 20 × objective (Plan-Apochromat 20x/0.8 M27), using identical settings. Analyses and quantifications were done in Zen software (2.6, Blue Ed.). For quantifications, we used a series of sections covering the rostrocaudal extent of EC from each of three animals, and double-immunolabeled against reelin and Bnip3. While visualizing only the channel used to scan the reelin signal (546-channel), we selected cell profiles using the Zen Circle Tool, taking care to obtain good coverage also of the rostrocaudal axis, but avoiding the part at the ventral extreme (i.e. the part furthest away from the rhinal fissure) since the signal here is very weak relative to background. After this selection, we switched to the channel for the Bnip3 signal (488-channel) and identified each circle with a clearly positive signal. By this we obtained the ratio between Bnip3-positive neurons and reelin-positive neurons, i.e. $\frac{\# Bnip3\:positive\:neurons}{\# reelin\:positive\:neurons}$.

**Fig. 1:**
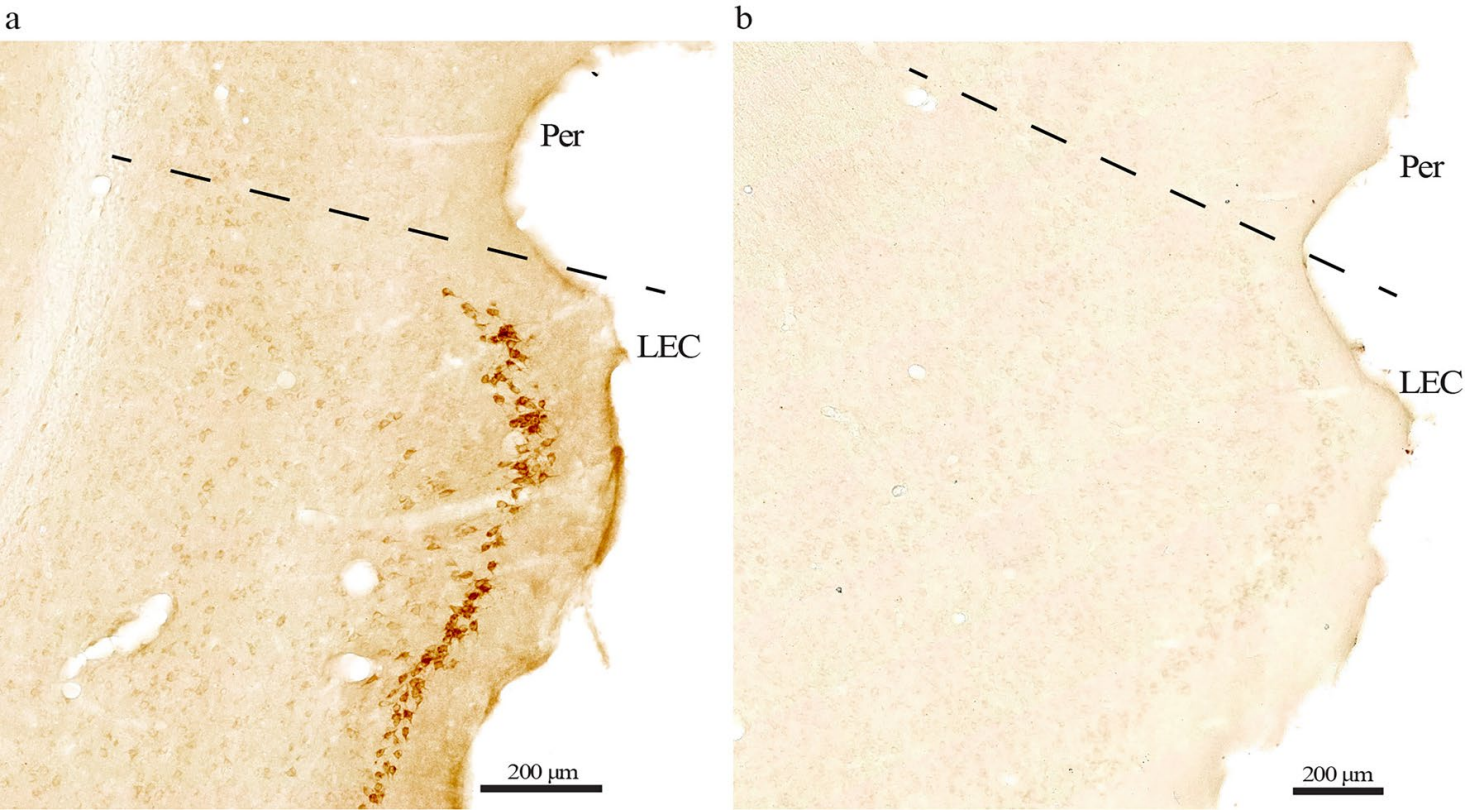
No evident background labeling in our setup. **a** Immunolabeling with the Bnip3 antibody reveals a high level of signal in LEC layer II of Wistar rats (example from 18-month-old animal). **b** Running the same experiment without the Bnip3 antibody while keeping all else equal shows a complete absence of signal. The same is the case across the age groups. Scale bars indicated on each figure. Dashed line marks border between LEC and the perirhinal cortex (Per)

*Controlling for background labeling by the secondary antibodies.* To control for possible background labeling by the secondary antibodies we immunolabeled sections containing EC from different age groups and omitted the primary antibody in one section per run. We found no signs of background labeling in our setup (Fig. 1).

**Fig. 2:**
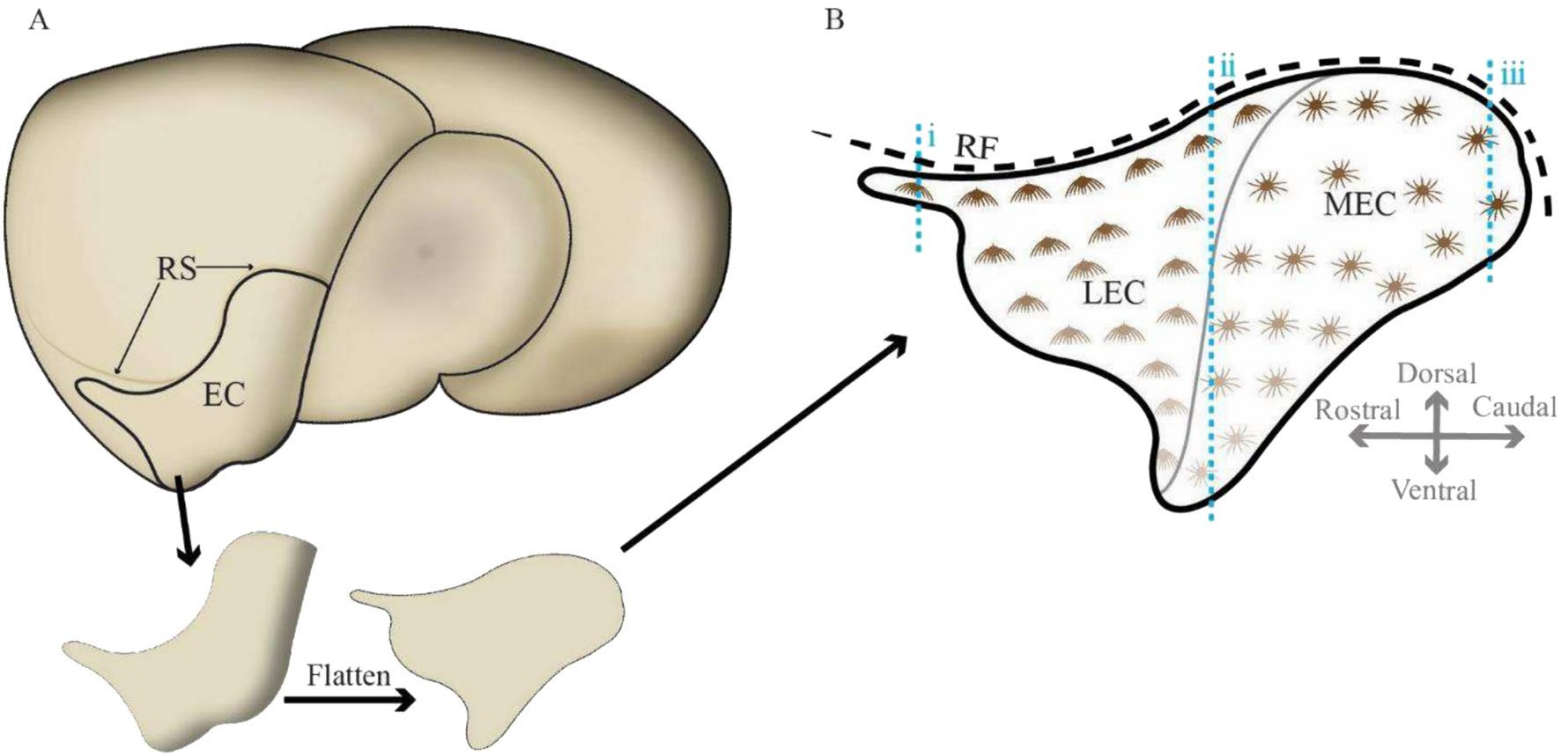
Guide to orientation of sections. **a** Cartoon of rat brain seen from a laterocaudal perspective, with the situation of the entorhinal cortex (EC) indicated, and an illustration of a virtual resection and compression of EC into a flatmap. **b** Matching flatmap of EC layer II with reelin-expressing fan cells illustrated for the lateral entorhinal cortex (LEC) and reelin-expressing stellate cells illustrated for the medial entorhinal cortex (MEC). The decrease in color intensity for the cells indicate the decrease in reelin-expression as one moves successively away from the rhinal fissure (RF). Blue dashed lines (i–iii) indicate the locations of the coronal sections displayed in Fig. 3a–c, respectively

*Orientation of sections*. To aid visualisation of the position of the sections shown in the Results, we provide a cartoon (Fig. 2).

### Bnip3 expression in the entorhinal cortex

**Fig. 3:**
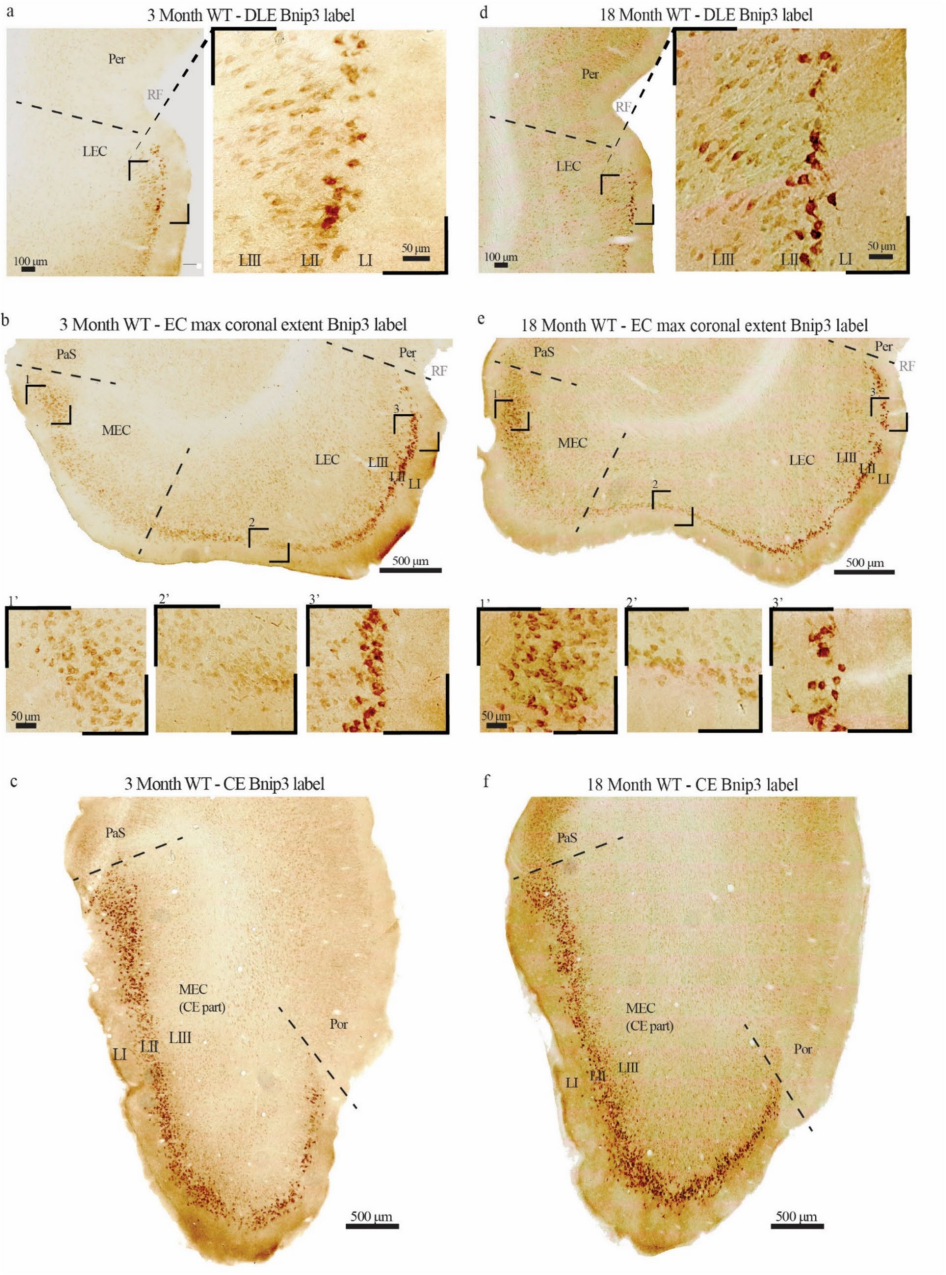
A major subset of ECLII neurons expresses Bnip3. **a** Example of the dorsolateral EC (DLE, i.e. the anterior tip of LEC), in which LII-neurons are particularly strongly stained. **b** The expression of Bnip3 gradually decrease at positions successively further away from the rhinal fissure (RF), irrespectively of whether one is located in LEC or MEC (see Fig. 1 as a guide to how the sections relate to each other across EC and between LEC vs MEC). **c** Example at the caudal extreme (CE) of EC where only the part of MEC located close to the rhinal fissure is present in coronal sections. (**d–f**) shows the corresponding labeling for 18 month old animals. *Per* Perirhinal cortex, *PaS* Parasubiculum, *Por* Postrhinal cortex. Scale bars are indicated for each figure, note that for the insets in (**b**) and (**e**) the scale bar in the leftmost inset applies to all

We found that the expression of Bnip3 is near undetectable in the cell somata of most parts of the forebrain. This is in line with previous reports (He et al. 2019; Lu et al. 2014; Zhang et al. 2011; Althaus et al. 2006) and the finding that pathological conditions such as hypoxia or ischemia are required to trigger increased expression (Lu et al. 2014; Zhang et al. 2011; Althaus et al. 2006). However, in stark contrast to this general finding, a major subset of neurons in the part of EC LII that is located close to the rhinal fissure (i.e., dorsally) expressed high levels of Bnip3 in all age groups (Fig. 3 and Supplementary Fig. 1). Since the only difference between young and old rats appears to be a modest overall increase in staining intensity with age, the description to follow holds true for all age groups. The expression of Bnip3 in both LEC and MEC manifests a striking gradient (Fig. 3b and c). While the expression is high in LII-neurons located close to the rhinal fissure, the expression level gradually decreases at positions successively further away from the rhinal fissure, to the point where the level in the most ventrally located LII-neurons is almost below the detection limit.

### Bnip3-expression is restricted to the reelin-positive ECLII-population

Assuming that the level of Bnip3 expression serves as a proxy of the neuronal mitochondrial turnover rate, and that the turnover rate is positively correlated with the neuronal metabolic rate, it follows from the data (Fig. 3) that the overall neuronal metabolic rate is substantially higher in EC LII than in other layers of the EC, as well as in most other parts of the rat brain (see below). And the data clearly suggest that energy expenditure is highest in neurons located close to the rhinal fissure.

**Fig. 4:**
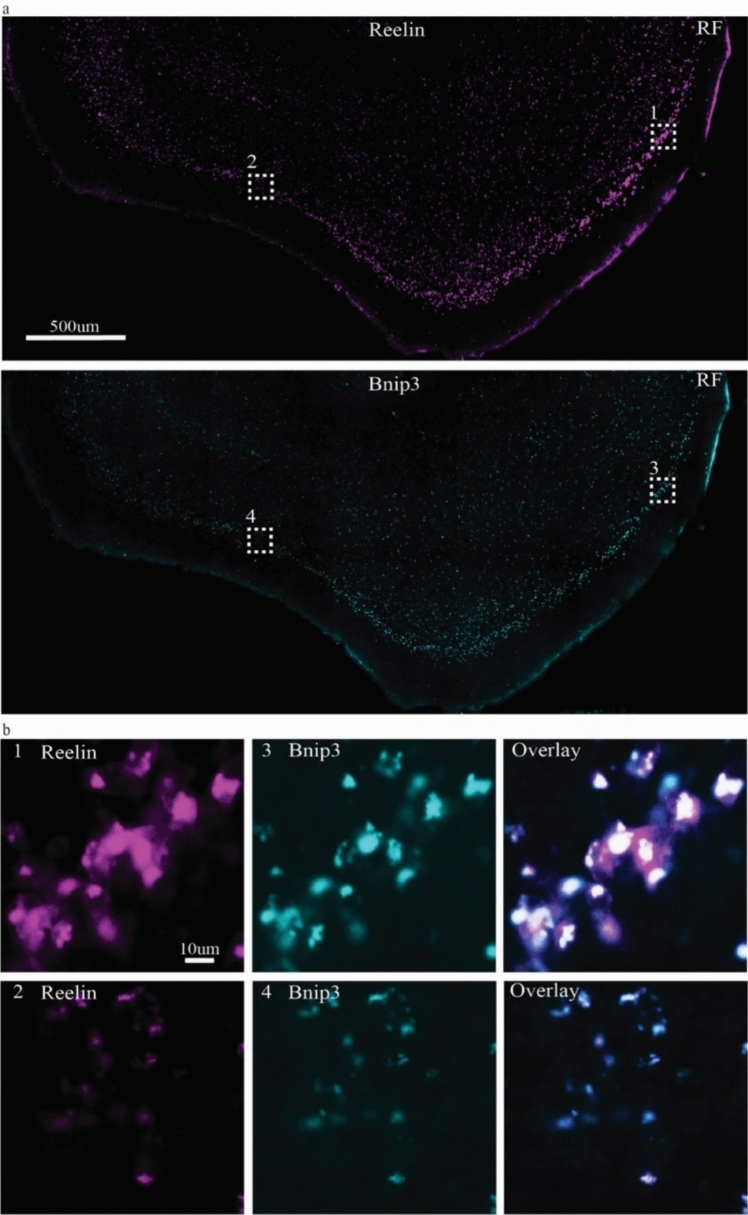
Double-immunolabeling against reelin and Bnip3. **a** Top panel shows labeling for reelin, bottom panel shows labeling for Bnip3. **b** Higher powered insets (numbers correspond to those in (**a**)) reveal that Bnip3 follows the same gradient as reelin, thus being strong towards the rhinal fissure (RF) and increasingly less so as one moves successively further away from the rhinal fissure. Further, it is apparent from these images that Bnip3 is confined to the population of Re + ECLII neurons. Scalebar for **a** applies to both images; scalebar for **b1** applies to all six images

Since the strength of the Bnip3 signal clearly appeared to align with the previously reported reelin expression gradient in ECLII (Kobro-Flatmoen et al. 2016), we double-immunolabeled against reelin and Bnip3. The results (Fig. 4) clearly suggest that high Bnip3 expression is restricted to the Re + ECLII neurons.

We therefore took one series of sections from each of three animals and quantified the degree of co-localization between Bnip3 and reelin throughout ECLII. Applying stringent criteria for what qualifies as a positive Bnip3 signal, we found that Bnip3 expression was restricted to Re + ECLII neurons, and that approximately 90% of Re + ECLII neurons were positive for Bnip3 ($\frac{Bnip3\:positive\:neurons}{reelin\:positive\:neurons}$; animal 1$: \frac{607}{688}=0.88$; animal 2:$\frac{629}{691}=0.91$; animal 3:$\frac{699}{774}=0.90$). This is probably a conservative estimate, and we expect that more extensive analyses are likely to find that the degree of overlap is even higher. Given that the Bnip3 expression level serves as a proxy for neuronal metabolic rate, it appears that the data support our prediction (see Introduction) that the observed reelin-gradient in ECLII reflects the overall energy expenditure of the neurons along this gradient. To visually demonstrate that Bnip3 is specifically co-localized with Re + ECLII neurons and hence does not co-localize with Cb + ECLII-neurons, we performed triple-immunolabeling experiments against Bnip3, reelin and calbindin. These experiments further demonstrate the selective co-localization of Bnip3 with Re + ECLII neurons (Supplementary Fig. 2).

### Indication that Bnip3 may be expressed also in human EC LII neurons

**Fig. 5:**
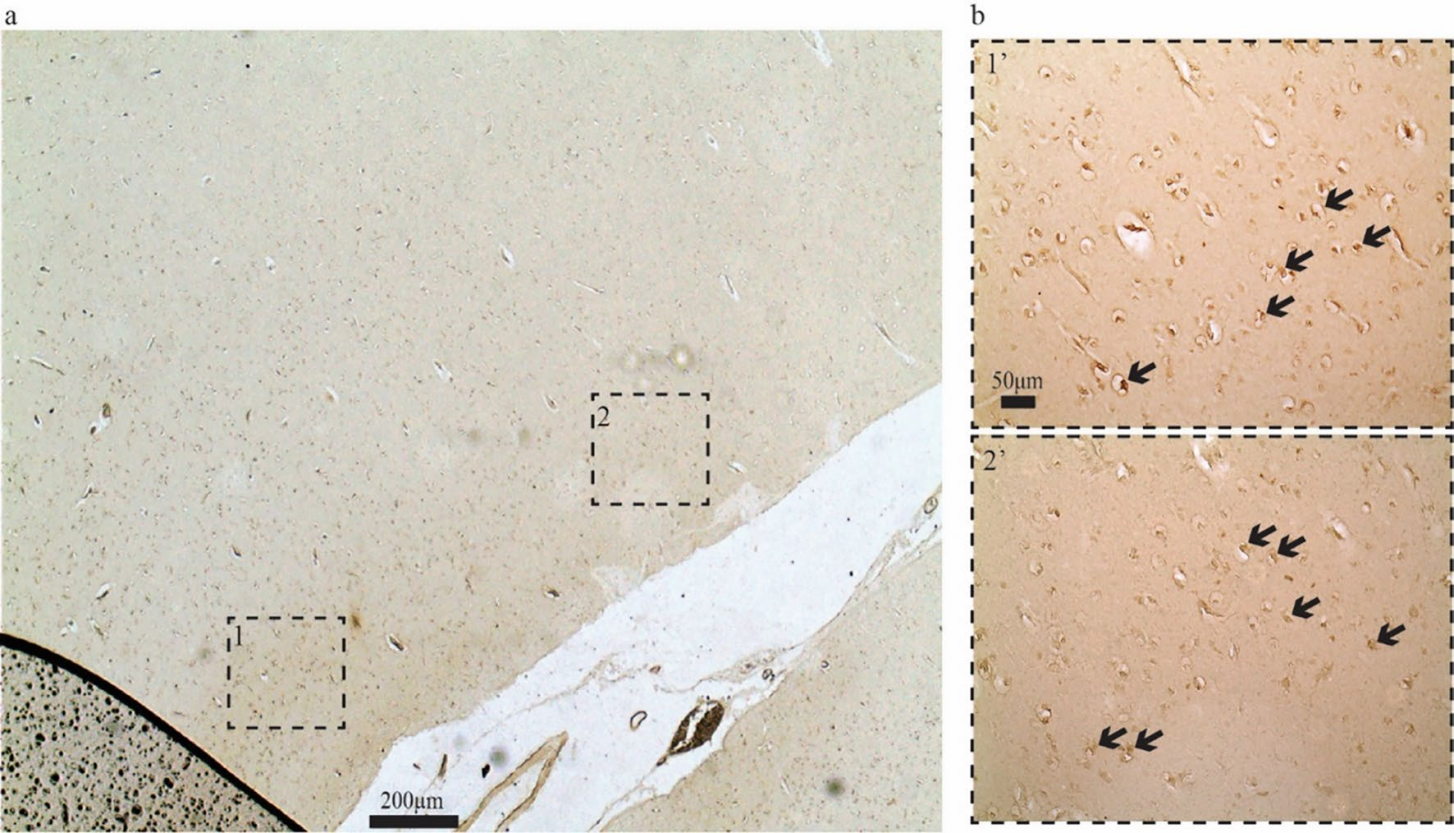
Bnip3-expression in human ECLII neurons. **a** Immunolabeling against Bnip3 in EC of a human subject appears to reveal a signal that is **b** most notable in LII (the insets in (b) match those in (**a**)). Arrows in (**b**) indicate examples of what are likely positive neurons. Scale bars are indicated in figures, (**b** **1’**) also applies to (**b** **2’**)

In a pilot test, we also found Bnip3-expression in human ECLII neurons (Fig. 5). But since we do not currently have access to complete human EC samples, we were prevented from assessing whether all our results obtained in Wistar rats extend to humans. However, as in rats, reelin is expressed in human principal ECLII neurons following the same topographical gradient (Kobro-Flatmoen and Witter 2019). Therefore, we consider it likely that a close overlap between reelin expression and Bnip3 expression will also be the case in humans.

### Other neurons expressing elevated levels of Bnip3 in rats

**Fig. 6:**
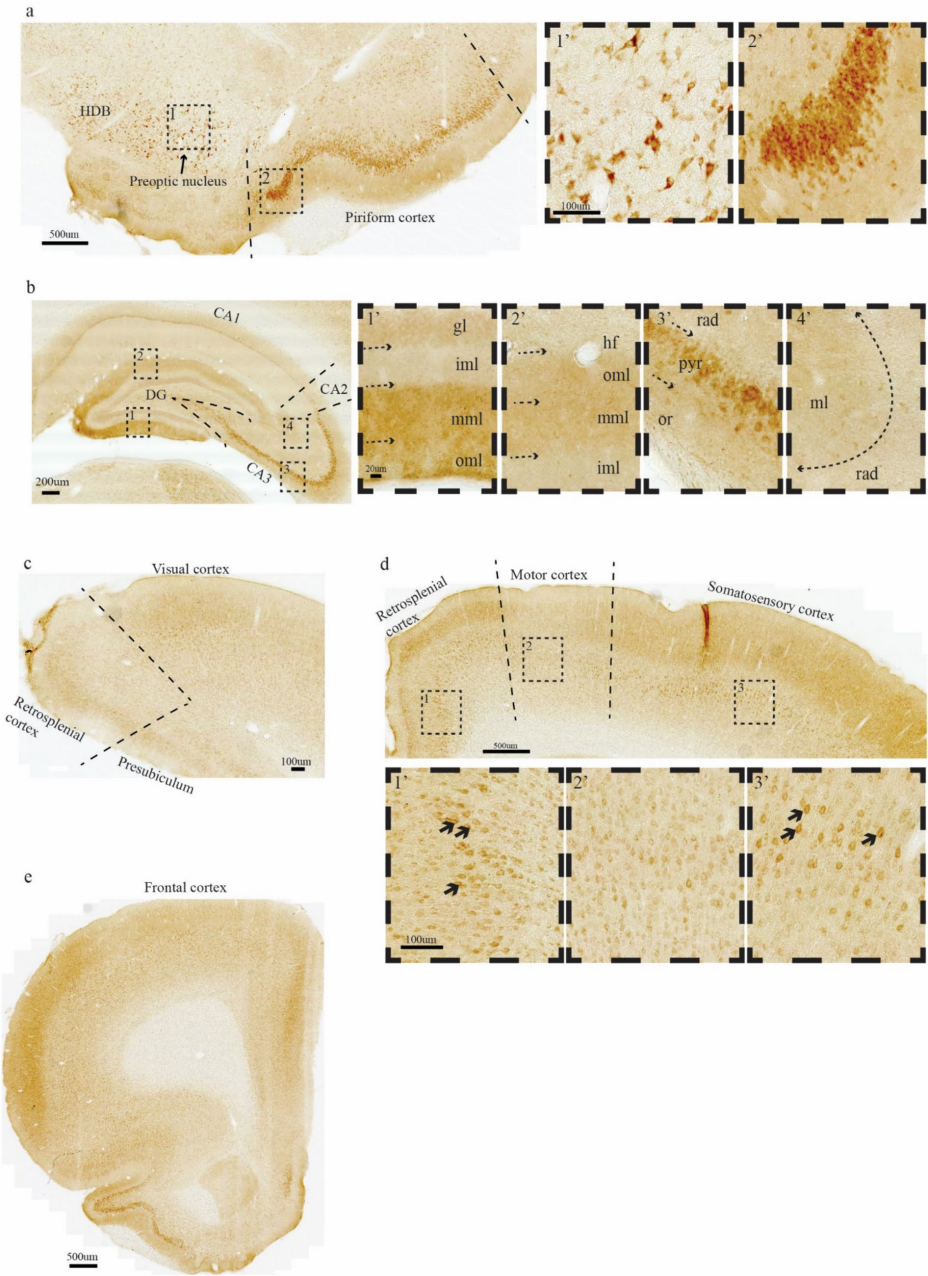
Moderate levels of Bnip3-expression is present in certain structures outside of the entorhinal cortex. This includes **a** neurons in the piriform cortex and the preoptic nucleus, **b** CA3 (note the neuropil labeling in the outer 2/3ds of the molecular layer of DG, such labeling is also present in CA3 and CA2, albeit to a lesser degree as is clear from the insets), **c** visual cortex, **d** retrosplenial-, motor-, and somatosensory cortex, and **e** frontal cortex. Scale bars are indicated in each figure, note that for the related insets, the leftmost scale bar applies to all. **a** *HDB* Nucleus of the horizontal limb of the diagonal band, **b** *gl* granular layer of dentate gyrus, *iml* inner molecular layer of dentate gyrus, *mml* middle molecular layer of dentate gyrus, *oml* outer molecular layer of dentate gyrus, *hf* hippocampal fissure, *rad* stratum radiatum, *pyr* stratum pyramidale, *or* stratum oriens

We then used Bnip3 as a probe to identify neurons or neuronal clusters that putatively have higher than average mitochondrial activity (Fig. 6). LII-neurons of the piriform cortex and occasional neurons with high levels in the preoptic nucleus display moderate and high levels of Bnip3 expression, respectively (Fig. 6a). In the hippocampal formation, the expression of Bnip3 is generally low or absent, except for field CA3, where a substantial number of neurons in the pyramidal layer express moderate levels. Notable levels of Bnip3-expression is also found in the outer 2/3ds of the molecular layer of the dentate gyrus (DG) and in stratum lacunosum moleculare of CA3/2, which contains the Re + ECLII neuron-terminals; we also observe that there is a tendency for more intense labeling in the free (lower) blade of DG (Fig. 6b). Furthermore, in parts of the visual cortex (Fig. 6c), the retrosplenial, somatosensory and motor cortices (Fig. 6d), and the frontal pole (Fig. 6e) we find subsets of neurons where Bnip3 expression is clearly above background, sometimes reaching moderate levels.

**Fig. 7:**
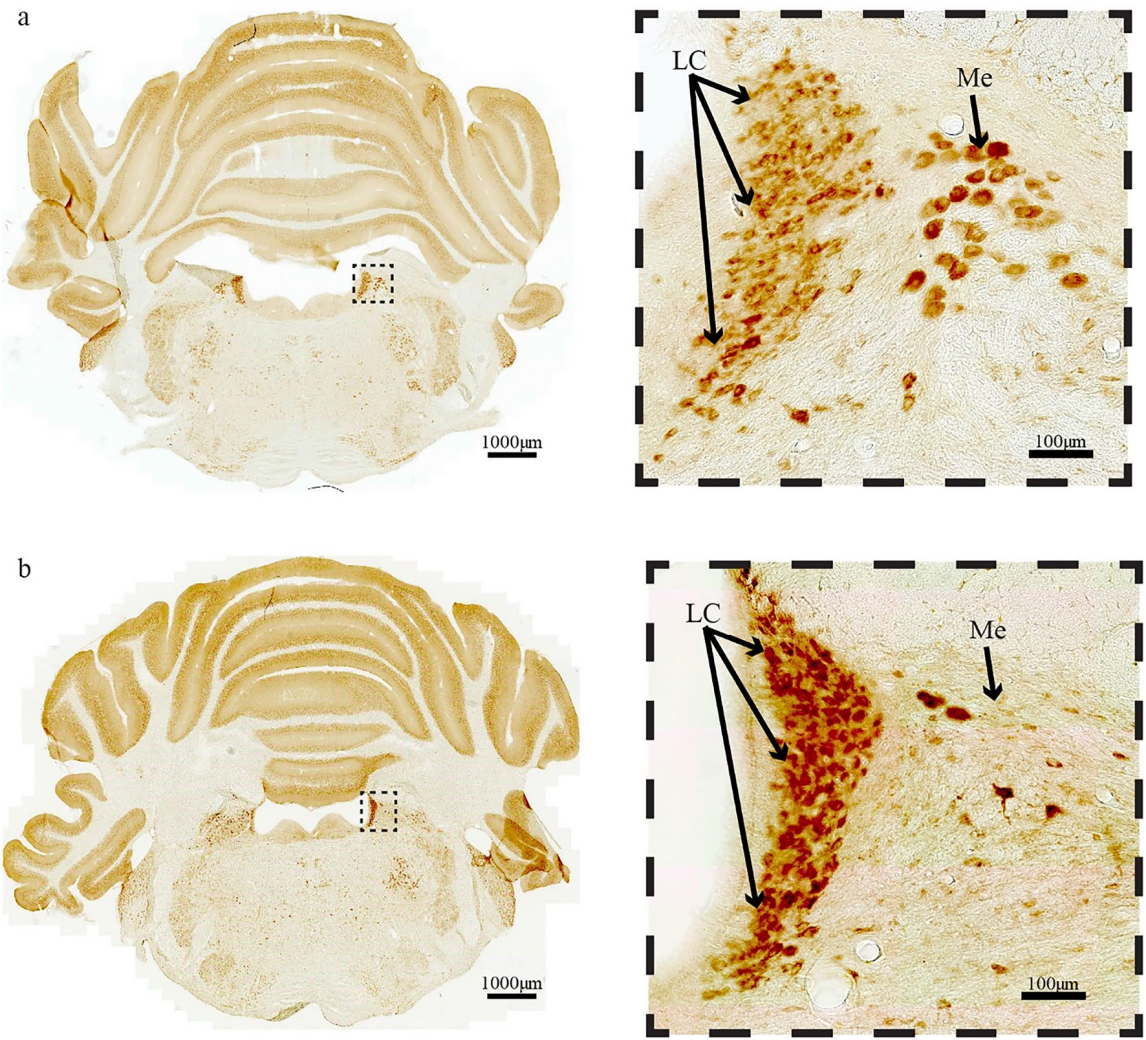
High levels of Bnip3-expression in neurons of the locus coeruleus (LC) shown in **a** a three month old rat and **b** an 18 month old rat. Equally high levels are found in a subset of neurons belonging to the mesencephalic trigeminal nucleus (ME). Scale bars are indicated in each figure

**Fig. 8:**
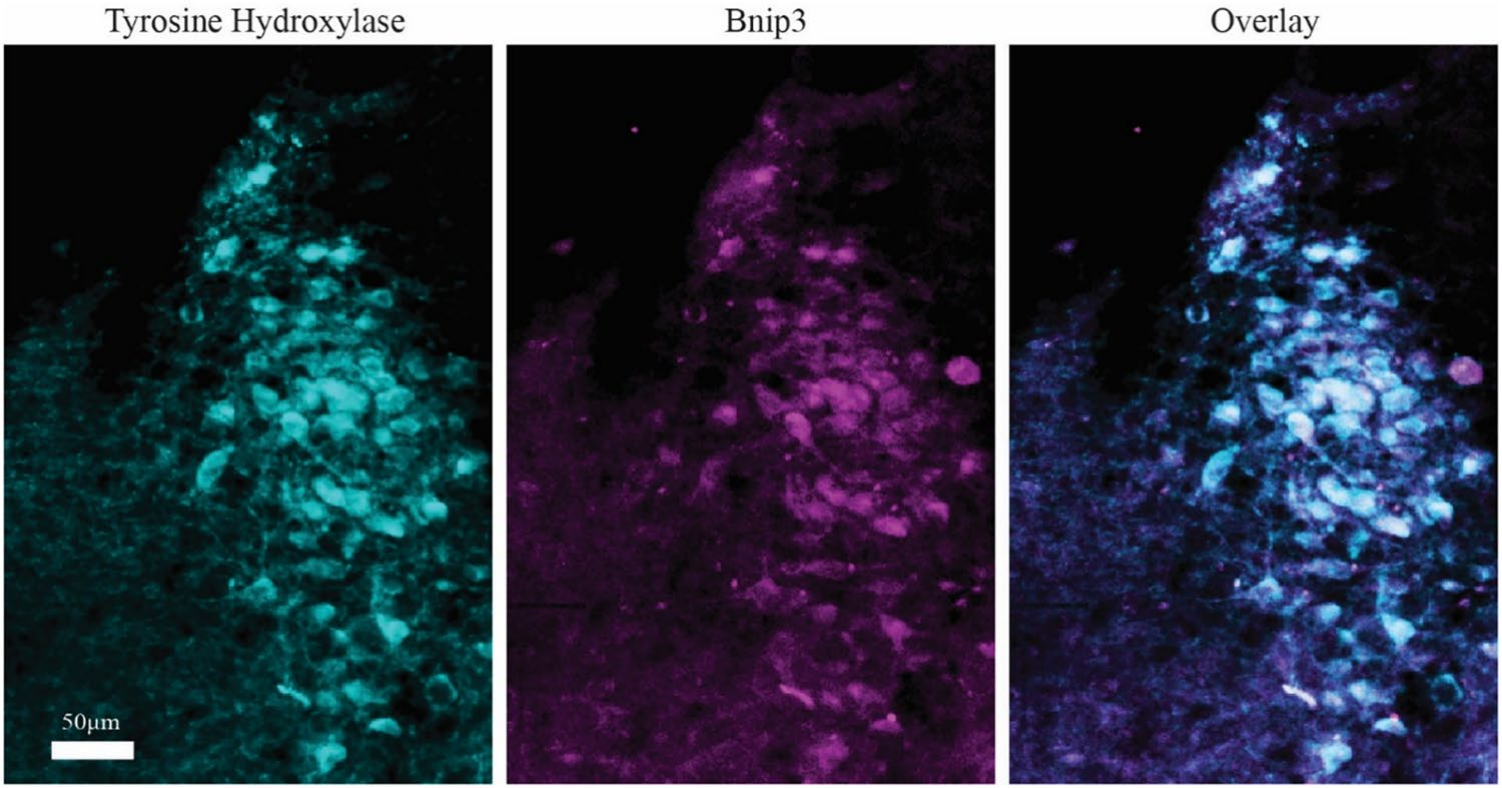
Bnip3-expression in neurons of LC co-localize with tyrosine hydroxylase. Scale bar in leftmost image applies to all

Bnip3-expression in neurons of the dorsomedial part of the locus coeruleus (LC) is also high (Fig. 7a, b). We confirmed that these Bnip3 high-expressing neurons are indeed part of LC by double-labeling against tyrosine hydroxylase (Fig. 8). The moderate to high Bnip3 expression found in non-Re + ECLII neurons suggests that these neurons also have higher than average metabolic rate, but it is beyond the scope of this paper to elaborate on the reasons for this.

## Discussion

Re + ECLII neurons are known to have compromised mitophagy from the earliest stages of AD (Fang et al. 2019; Kerr et al. 2007). Our results are relevant in this context, as they clearly support that, compared to most other neurons, Re + ECLII neurons generally have a particularly high energy requirement. And because the size of the standing pool of mitochondria is homeostatically regulated under normal conditions (Kowald and Kirkwood 2018), enhanced Bnip3 expression also implicates enhanced mitogenesis. Therefore, the replenishment of the mitochondrial pool might be affected by modulation of Bnip3 expression in the very early stages of AD development.

The observed expression gradient of Bnip3 indicates that although the metabolic rate in Re + ECLII neurons is generally high, it varies substantially in a systematic way. A systematic reduction in metabolic rate as one moves away from the rhinal fissure is in line with the distribution of the mitochondrial enzyme cytochrome oxidase in EC (Hevner and Wong-Riley 1992). We propose that the reason why Re + ECLII neurons display the reelin and Bnip3 gradients is tied to the level of spatial and temporal detail by which they encode information about the external environment. Most of the entorhinal input to the hippocampal formation arises from layers II and III, and the Re + ECLII neurons make up the exclusive layer II population that projects to the dentate gyrus and the CA3/2 hippocampal fields (Varga et al. 2010). In rodents, EC-neurons located close to the rhinal fissure are more strongly connected to the dorsal part of the hippocampal formation, while successively more ventrally situated EC neurons are most strongly connected to correspondingly more ventral levels of the hippocampus (Kobro-Flatmoen and Witter 2019; Canto et al. 2008). Intriguingly, the level of detail by which spatial information about the external environment is encoded is highest at the dorsal extreme of the hippocampus (or the corresponding posterior extreme in humans) and gradually decreases as one moves ventrally (or anteriorly in humans) (Evensmoen et al. 2015; Kjelstrup et al. 2008; Brun et al. 2008). Arguably, the more detailed the encoded environmental representation, the faster it will become outdated. Thus, to keep track of changes in the spatial environment, high-resolution representations are putatively generated and modified by the entorhinal-hippocampal system much more frequently than low-resolution ones. This conception is consistent with data on the only type of entorhinal neuron for which we know the level of detail encoded, namely the grid cells in MEC, whose firing form a representation of space by tessellating the environment explored by the animal to form a hexagonal grid pattern (Hafting et al. 2005). Thus, grid cells with the most detailed grid pattern sit close to the rhinal fissure, while the grid cells with increasingly less detailed grid patterns are located increasingly farther away from the rhinal fissure (Stensola et al. 2012). It is reasonable to expect that the need to update representations of the environment follows the scale under consideration. For example, several relevant details will change between the different times a rodent traverses a given stretch in its natural habitat, but the distal landmarks remain the same.

On the assumption that levels of detail will prove to be encoded along the same gradient also for other, non-spatial representations of the environment, such as those of time or event sequences (Tsao et al. 2018), and objects (Tsao et al. 2013), we anticipate that the graded expression levels of reelin and Bnip3 will turn out to be directly related to the frequency with which it is necessary to update environmental representations in the broad sense. Since reelin is a canonical synaptogenic protein (Qiu et al. 2006; Rogers et al. 2011) heavily involved in the remolding of synaptic contacts (Wasser and Herz 2017), it makes at least perfect sense that its expression is highest in those neurons where the environmental representations are most frequently updated. And since this remolding must necessarily also be energetically demanding, this appears to explain why the Bnip3 expression is so closely tied to the reelin expression in Re + ECLII neurons.

## Supplementary Information

Below is the link to the electronic supplementary material.

Supplementary file1 (jpg 2983 KB)

Supplementary file2 (jpg 6071 KB)

## Data Availability

Raw data will be made available upon request.
