# Correction: Bnip3 expression is strongly associated with reelin‑positive entorhinal cortex layer II neurons

**DOI:** 10.1007/s00429-025-02967-9

**Published:** 2025-07-03

**Authors:** Stig W. Omholt, Raissa Lejneva, Maria Jose Lagartos Donate, Domenica Caponio, Evandro Fei Fang, Asgeir Kobro‑Flatmoen

**Affiliations:** 1 Department of Circulation and Medical Imaging, Norwegian University of Science and Technology (NTNU), 7491 Trondheim, Norway; 2 Kavli Institute for Systems Neuroscience, Norwegian University of Science and Technology (NTNU), 7491 Trondheim, Norway; 3 K. G. Jebsen Centre for Alzheimer’s Disease, Norwegian University of Science and Technology (NTNU), 7491 Trondheim, Norway; 4 Department of Clinical Molecular Biology, University of Oslo and Akershus University Hospital, 1478 Lorenskog, Norway

**Correction: Brain Structure and Function (2024) 229:1617–1629** 10.1007/s00429-024-02816-1

Following publication of the original article, the author identified an error in Fig. 1(a, b)

For completeness and transparency, the old incorrect versions are display below
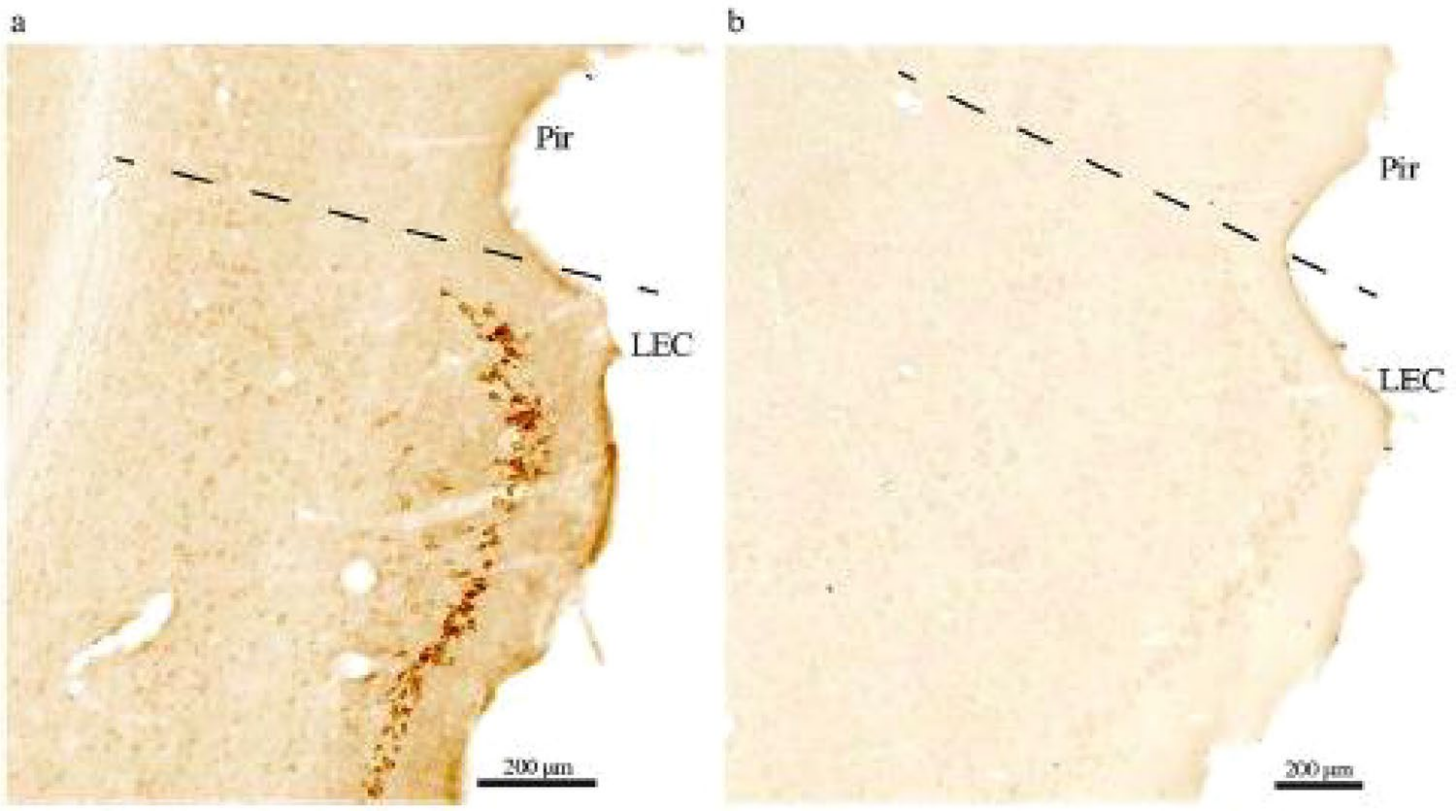


The Corrected Version of Fig. 1(a, b)
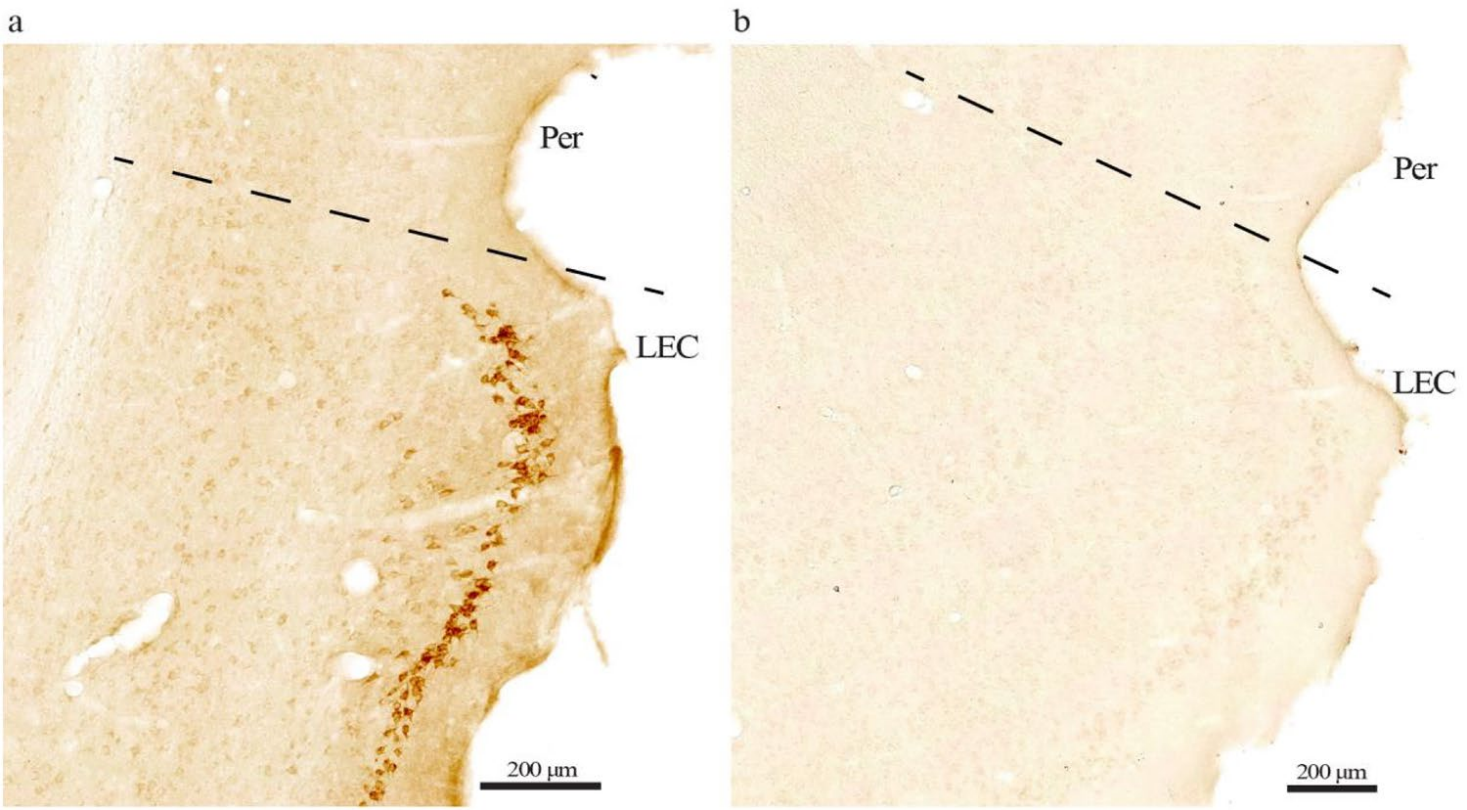


In the sentence beginning of Fig. 1 No evident background labeling in our setup… **piriform cortex (Pir)** should have read as **“perirhinal cortex (Per)”.**

In the sentence beginning of Fig. 3, A major subset of ECLII neurons expresses… **Postsubiculum** should have read as **“Postrhinal cortex”**.

