# Supplementary figures and images for: Bnip3 expression is strongly associated with reelin-positive entorhinal cortex layer II neurons
Source: Brain Struct Funct. 2024 Jun 25;229(7):1617–29. doi: 10.1007/s00429-024-02816-1 (PMC11374853; doi:10.1007/s00429-024-02816-1)

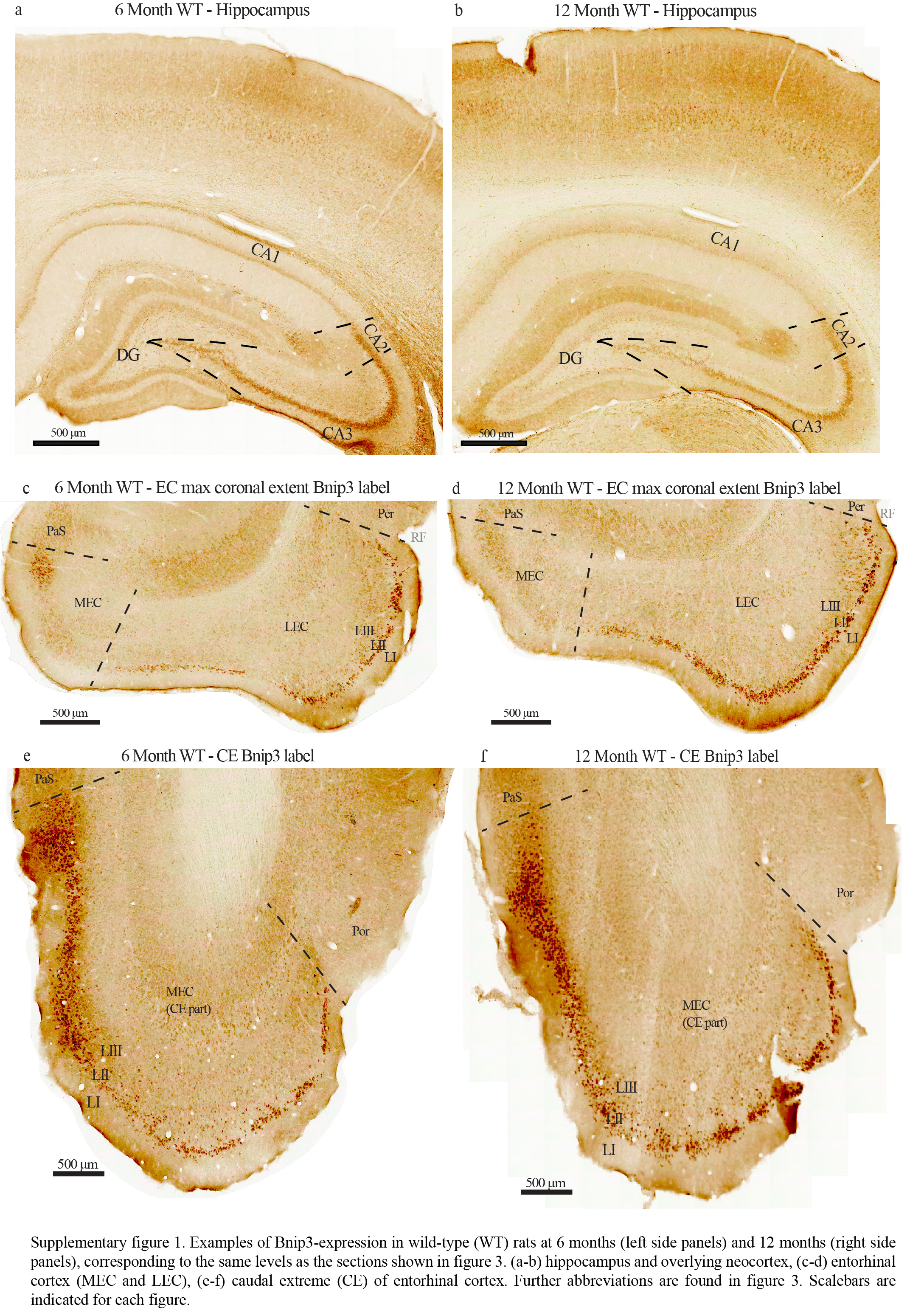

Supplement: Supplementary file 1 — Supplementary file1 (jpg 2983 KB) [file 429_2024_2816_MOESM1_ESM.jpg]

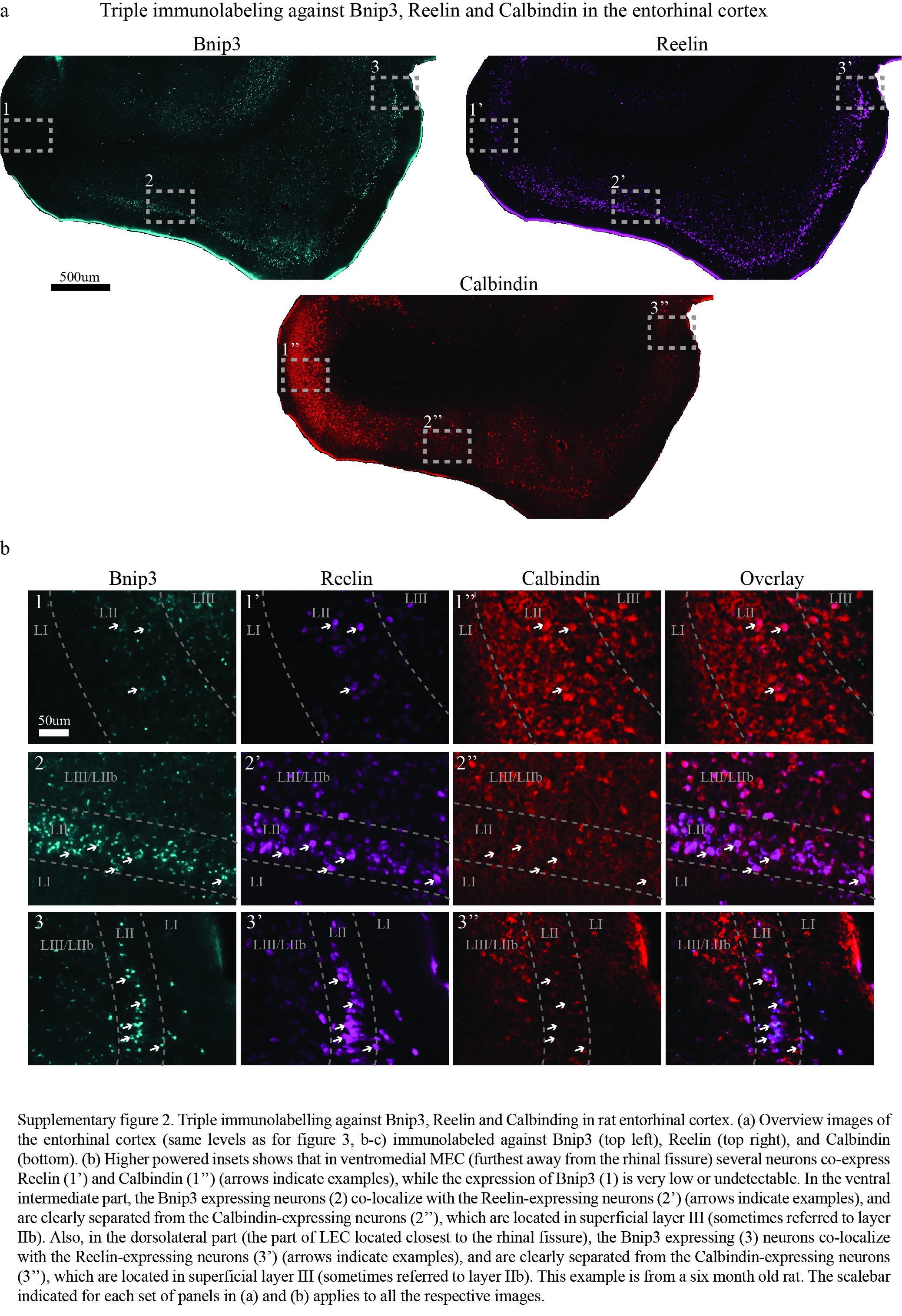

Supplement: Supplementary file 2 — Supplementary file2 (jpg 6071 KB) [file 429_2024_2816_MOESM2_ESM.jpg]
